# From food to alcohol: exploring psychosocial drivers of post-bariatric alcohol use

**DOI:** 10.1080/17482631.2026.2632736

**Published:** 2026-02-27

**Authors:** Elizabeth Sharpe, Daniel Frings, Kerry Wood

**Affiliations:** a School of Applied Sciences, London South Bank University, London, UK

**Keywords:** Bariatric surgery, alcohol use, self-medication, trauma, qualitative research

## Abstract

**Background:**

Bariatric surgery is an effective intervention for severe obesity; yet emerging evidence indicates a clinically significant risk of problematic alcohol use post-operatively, particularly among women and those undergoing Roux-en-Y gastric bypass. While physiological mechanisms are well documented, , limited qualitative research explores the psychosocial drivers behind this shift.

**Methods:**

Using reflexive thematic analysis, we examined semi-structured interviews with 11 UK-based women (aged 33–63) who underwent bariatric surgery (2012–2021) and subsequently developed problematic alcohol use, despite no formal diagnosis of alcohol use disorder. Interviews were co-designed with individuals with lived experience and thematically coded in NVivo.

**Results:**

Five themes emerged, each comprising related sub-themes: (1) altered alcohol metabolism and heightened intoxication; (2) alcohol as a substitute for food and tool for weight control; (3) shifting identity and social confidence; (4) alcohol use as emotional regulation post-weight loss; and (5) insufficient pre- and post-operative psychosocial care. Participants described using alcohol deliberately for appetite suppression, social ease, or emotional escape, reflecting unmet psychological needs.

**Conclusions:**

Post-surgical problematic alcohol use appears not simply physiological but a coping response shaped by trauma, identity change, and unmet emotional needs. Trauma-informed screening and longitudinal psychosocial follow-up are needed to improve long-term outcomes.

**What is already known**

- Bariatric surgery has been associated with an increased risk of problematic alcohol use, particularly following Roux-en-Y gastric bypass, with emerging evidence also suggesting elevated risk following sleeve gastrectomy, albeit to a lesser extent.
- Addiction transfer from food to alcohol has been reported but remains poorly understood in psychosocial terms among bariatric surgery patients.
- Current care pathways still focus mainly on physical outcomes, with less attention to psychosocial risks.

**What this study adds**

- Shows how alcohol is used for appetite suppression, social confidence, and emotional coping after surgery.
- Demonstrates how post-operative challenges and social dynamics can escalate alcohol use.
- Underscores the need for improved assessment, patient education, and tailored psychosocial support in bariatric care.

## Introduction

Alcohol consumption poses serious health risks, including liver disease, cardiovascular issues, psychiatric disorders, and broader societal costs (Cargiulo, 2007; Room et al., 2016). These risks are especially concerning for vulnerable groups, such as individuals undergoing bariatric surgery for obesity, a condition linked to comorbidities like diabetes, cardiovascular disease, and psychological distress (Frank et al., 2022). While bariatric surgery offers sustained weight loss and reduced health risks (Sjöholm et al., 2025) emerging evidence highlights a concerning post-operative complication: alcohol use disorder (AUD).

AUD prevalence increases significantly after bariatric procedures, particularly Roux-en-Y gastric bypass (RYGB), with rates rising from 4.5% pre-surgery to as high as 28.4% post-surgery (King et al., 2012; Leggio et al., 2025; Suzuki et al., 2012). Individuals undergoing RYGB are more likely to develop AUD than non-surgical controls and experience higher rates of alcohol-related hospital admissions compared with those receiving restrictive procedures (Östlund et al., 2013; Svensson et al., 2013). Evidence further suggests that post-operative alcohol-related risk varies by bariatric procedure, with the strongest associations reported following Roux-en-Y gastric bypass, emerging evidence for sleeve gastrectomy, and less consistent findings for restrictive procedures; notably, sleeve gastrectomy is now the most commonly performed bariatric surgery in the UK (King et al., 2012; Östlund et al., 2013). More recent studies using different screening approaches and follow-up periods report lower prevalence estimates of post-operative problematic alcohol use than earlier cohorts, though rates vary considerably by assessment method, timing, and clinical context. Collectively, this literature indicates a clinically meaningful elevated risk rather than a uniformly high or rising incidence (Adeniran et al., 2025; Esin et al., 2025; Riedel et al., 2024).

In the UK, guidance on alcohol use following bariatric surgery has evolved over time and has varied across services, often focusing on short-term abstinence rather than longer-term risk. As participants in this study underwent surgery between 2012 and 2021, some may have received their surgery before clearer or more consistent post-operative guidance was routinely emphasised.

Proposed mechanisms underlying post-surgical alcohol risk include physiological changes (e.g., faster alcohol absorption), psychological vulnerabilities (e.g., unresolved trauma, emotional distress), and the concept of addiction transfer, where food dependence is replaced by alcohol (Brunault et al., 2015; Yoder et al., 2018). Increased socialisation and confidence following weight loss can also lead to heavier alcohol use. Although alcohol consumption may initially decline after surgery, longitudinal evidence suggests that use often increases within one to two years, underscoring the need for sustained post-operative support (Brunault et al., 2015; Svensson et al., 2013).

Evidence suggests that pre-operative substance use does not negatively affect weight loss outcomes, though it is linked to increased risk of post-operative substance use (Yoder et al., 2018). Despite growing clinical concern, much of the existing literature has relied on quantitative designs, with comparatively limited attention to patients’ lived experiences. Qualitative research is well positioned to explore how individuals understand, justify, and manage alcohol use in the context of life after bariatric surgery. This study examines the psychosocial drivers of post-operative alcohol use among women.

Women represent a substantial proportion of bariatric surgery recipients and may experience distinct vulnerabilities related to body image, emotional eating, trauma exposure, and affect regulation (Davy et al., 2006; Erol & Karpyak, 2015). Although epidemiological studies often report higher post-operative alcohol risk among men, focusing on women in this qualitative study enabled in-depth exploration of gendered psychosocial processes rather than prevalence comparisons.

While previous qualitative studies have examined patient perceptions of alcohol use following bariatric surgery, less attention has been paid to women’s lived experiences of *intentional* alcohol use as a coping strategy, including its role in appetite regulation, weight control, trauma-related coping, and identity change over time. Addressing this gap may inform more gender-sensitive, patient-centred approaches to post-operative care.

## Materials and methods

### Participants

Eleven UK-based female participants aged 33 to 63 years, who had undergone bariatric surgery 2 to 10 years prior (ages at surgery ranged from 28 to 55 years). Procedures included gastric bypass (5 participants), gastric sleeve (3), gastric band (2), and gastric sleeve followed by gastric bypass (1), conducted via the NHS (2), privately in the UK (7), or in Turkey (2). Participants were included regardless of bariatric procedure type, as the study aimed to explore psychosocial processes associated with post-surgical alcohol use rather than to compare procedure-specific risk profiles. All participants reported that their relationship with alcohol worsened following surgery and that they had not received a formal diagnosis of AUD prior to surgery, based on self-report. However, several participants retrospectively described patterns of regular or heavy alcohol consumption before surgery that may not have been recognised as problematic at the time, either by themselves or within clinical assessments.

At the time of interviews, six were consuming alcohol and five were not. Pseudonyms were used to ensure anonymity. Detailed participant profiles are summarised in Table I.

**Table I.** Participant characteristics.

Pseudonym	Age	Ethnicity	Surgery (place)	Year of surgery	Highest Educ	Relationship status/children	Current alcohol status
Claire	55	White British	Sleeve (Private UK)	2012	Degree	Relationship/children	Sober 4 years
Debbie	33	White British	Sleeve (Private UK)	2018	GCSE	Single/Children	Sober 2 months
Denise	50	White British	Bypass (Private UK)	2012	Degree	Relationship/no children	Sober 7 months
Jennifer	48	White British	Sleeve (Private Turkey)	2021	GCSE	Single/children	Sober 6 months
Lucy	44	White British	Bypass (NHS UK)	2013	Degree	Single/Children	Drinking
Petra	52	White British	Gastric Band (Private UK)	2018	GCSE	Married/Children	Drinking
Julie	59	White British	Sleeve & Bypass (NHS UK)	2012 & 2013	GCSE	Married/Children	Sober 5 months
Amy	45	White British	Bypass (Private Turkey)	2020	GCSE	Married/Children	Drinking
Maria	42	White European	Bypass (Private UK)	2020	A'Level	Single/Children	Drinking
Olivia	46	White British	Gastric Band (Private UK)	2018	A’Level	Single/Children	Drinking
Tina	63	White British	Bypass (Private UK)	2015	Dip HE	Relationship/Children	Drinking

### Inclusion/exclusion criteria

UK-based females; undergone bariatric surgery more than two years prior; problematic alcohol use post-surgery, including those who had reduced or stopped drinking. Participants were excluded if they reported a current or prior formal diagnosis of AUD at the time of surgery, based on self-report during eligibility screening or were under complex mental health care.

Participants were recruited through purposive sampling via Facebook groups focused on alcohol use, bariatric surgery, and post-operative alcohol-related concerns. If interested in taking part they were asked to contact the research team for more information. No one declined or withdrew after giving informed consent.

### Procedure

Ethics approval was granted by the Division of Psychology Internal Ethics Committee at LSBU, ref: 16315559. Recruitment and data collection occurred in early 2023.

### Consent

Informed written consent was obtained from all participants before conducting interviews. Interviews were conducted via Zoom to accommodate geographical spread, in private settings selected by participants and audio recorded. Alongside interview data, sociodemographic details (e.g., age, relationship status, education, alcohol use) were recorded. Interviews were transcribed verbatim, anonymized, and assigned pseudonyms before being analysed. Field notes were recorded after each interview to capture contextual observations.

After approximately 10 hours of interviews with 11 participants, the dataset was judged to be sufficiently rich and diverse to meaningfully address the analytic aims. Decisions about sample size were guided by the depth, complexity, and variation of participants’ accounts, rather than a priori notions of saturation. Data collection ceased when additional interviews were contributing further nuance and elaboration, rather than generating substantially new conceptual insights relevant to the research questions.

### Interview schedule

The interview schedule was developed in consultation with individuals with lived experience of alcohol addiction or weight and food issues and informed by existing literature. Key areas explored included: (1) participants’ beliefs about the origins of food and alcohol problems; (2) self-perception of quality of life pre- and post-surgery; (3) habits, triggers, emotions, and stressors linked to food and alcohol use with comparisons to others (4) support. Interviews lasted 45–80 minutes and were conversational in tone, allowing flexibility to follow the participant’s narrative. All interviews were audio-recorded with participant consent.

### Data analysis

The interviews were analysed using reflexive thematic analysis, guided by Braun and Clarke’s (2006, 2019, 2021) six-phase framework. This approach facilitated an in-depth and iterative engagement with the data, enabling the development of interpretative codes and themes. Transcripts were read and re-read to support immersion, with initial observations documented in reflexive notes. Coding was primarily inductive and conducted using NVivo software, allowing patterns of meaning to be identified across the dataset. Both researchers involved in the analysis had formal training and prior experience in conducting reflexive thematic analysis, and analytic decisions were informed by ongoing reflexive engagement with the data and relevant methodological literature.

Researchers engaged with the data to support interpretative depth and reflexive dialogue, rather than to establish coding reliability or agreement. Initial coding focused on participants’ explicit accounts, with subsequent analytic discussions used to question assumptions, explore alternative interpretations, and develop more interpretive, latent themes. Disagreements were treated as analytically productive, prompting deeper engagement with the data rather than resolution through consensus.

Participant validation of transcripts or themes was not undertaken, as reflexive thematic analysis does not position member checking as a requisite indicator of analytic quality. Instead, credibility was supported through iterative, reflexive engagement with the data, collaborative analytic dialogue, and the co-design of the study with individuals with lived experience, consistent with reflexive qualitative practice.

Interviews were conducted by two female researchers, one with professional training and experience as a registered alcohol counsellor, which likely shaped rapport and facilitated disclosure on sensitive topics such as trauma and alcohol use. The researcher entered the study with clinically informed assumptions about alcohol substituting for food and increased tolerance post-surgery; however, analytic engagement challenged these expectations, particularly regarding alcohol use for weight control, limited post-operative follow-up, and under-recognised pre-surgical drinking.

Reflexivity was supported through analytic memos, team discussions, and iterative review of interpretations to ensure themes were grounded in participants’ accounts rather than prior assumptions.

Reporting of the study was guided by established qualitative reporting standards, including the Consolidated Criteria for Reporting Qualitative Research (COREQ) (Tong et al., 2007).

## Results

The analysis focused on identifying shared patterns of meaning and psychosocial processes across participants’ accounts, rather than on systematic comparison between subgroups (e.g., by surgery type or sobriety status). Given the small and heterogeneous sample, subgroup analysis was not undertaken, as this risked over-interpreting variation not consistently evident across the dataset.

Participants shared insightful narratives about their journeys leading up to bariatric surgery and their experiences with food and alcohol afterward. Their accounts can be categorised into five main themes. Table II provides a detailed overview of these themes and their sub-themes.

**Table II.** Themes and sub-themes.

Themes	Sub-themes
1. Altered physiology and accelerated alcohol sensitivity post-surgery	
2. Alcohol as a tool for weight control and appetitive management	Appetite suppression and/or food replacement
	Calorie decision—alcohol versus food
	Fear of weight regain leading to choosing alcohol instead
	Alcohol as a workaround for eating restrictions
3. Social dynamics of alcohol use: ego, image and relationships	Confidence, ego and social reinvention
	From eating to drinking: socialising after weight loss
	Friendship shifts around drinking
	Secrecy and social isolation
4. Alcohol as a mechanism for emotional coping and stress relief	Using alcohol as a coping mechanism
	Impact of adverse childhood experiences
5. Assessment, follow-up and peer support in alcohol related challenges.	Identify previous problematic alcohol use
	Assessment and follow-up
	Patients sharing personal experience
	Counselling post-surgery

**Theme 1: Altered physiology and accelerated alcohol sensitivity post-surgery**

Participants described significant changes in their alcohol tolerance and metabolism following surgery, leading to more rapid intoxication and subsequent challenges with dependency. Eight of the eleven participants highlighted the heightened effects of alcohol, often leading to unexpected consequences. Debbie remarked:

*“Definitely the tolerance changed, but the hits that I got from the drink... that was just crazy. I mean, everyone gets a little buzz-up from drink, but even from the get-go, the first drink after the surgery, like whoa... Yeah, it feels like you’ve done drugs or something.”*

Similarly, Maria shared how alcohol consumption was influenced by hunger and how the sensation alcohol gave her became addictive:

*“When I'm hungry, it's a real trigger for me to drink because I like the boozy rush on an empty stomach, and once I get that, I don’t want to eat, because I don’t want to lose that buzz.”*

Several participants recounted experiences where their altered tolerance led to heavier consumption and dangerous patterns of dependency. Jennifer recalled:

*“One time I felt awful…and I had some alcohol left over. I had some, and it made me feel better straight away. That's when it started. I knew I could drink the next morning, and I would feel better. And once I crossed that line, I couldn't go back.”*

Participants experienced heightened alcohol sensitivity and faster intoxication post-surgery, often leading to dependency. Many noted stronger effects from small amounts of alcohol, with some using it to cope with hunger or discomfort. This change in tolerance sometimes escalated into dangerous patterns, highlighting the risks associated with altered alcohol metabolism after surgery.

**Theme 2: Alcohol as a tool for weight control and appetitive management**

Weight control emerged as a significant theme, with participants highlighting the role of alcohol in managing calories from food or modifying eating behaviours to sustain weight loss post-surgery. This theme was further categorised into four sub-themes:

### Appetite suppression and food replacement

Participants highlighted how alcohol was used to suppress appetite or as a substitute for food. Julie reflected:

*“Food became less and less. Believe it or not, food—I ate very little because as my computer shut, I would have a glass of wine, and by the time my meal was ready, I didn’t really want to eat it, so I didn’t half the time. I just wasn’t interested really in eating when I’ve got a glass of wine in my hand, you know?”*

Similarly, Debbie shared how alcohol became an alternative to her previous eating patterns:

*“I replaced food with the alcohol in the way of the pattern, erm, so when I would have binged, I then started having a drink. But then, unfortunately, addiction has taken over, and it’s just spiralled. Spiralled past the pattern of how it was and when I would have been eating.”*

Olivia noted how drinking replaced meals:

*“I noticed I was drinking more because I couldn’t eat. So, I felt as if I was getting my energy or my calories or God knows, whatever, from that. Because I might not eat all day or night and just have a couple of drinks in the night, and I was fine. I was still functioning normal.”*

### Calorie trade-off: alcohol versus food

Participants discussed consciously choosing between consuming calories from alcohol or food, often prioritising alcohol to maintain their weight. For example, Olivia explained:

*“Knowing I could drink, and that it’s less calories than me eating. If I sat there with a bar of chocolate and a bag of sweets, I would probably put weight on. But by me having a couple of gins, I haven’t put any weight on. So that is probably a lot to do with it as well. Alcohol is high calories, but obviously food is more, isn’t it? It’s just replacing really, isn’t it? The alcohol isn’t putting weight on me whereas the food did.”*

Maria shared a similar perspective, emphasising her preference for alcohol over food:

*“I’ll think, oh, I’ve had 1000 calories in wine, and I best not have any tea... I’ll cook for the family and think I’ll just have a baby bel. It’s the calories. It’s like preference for booze than food. And I think if I eat, I won’t be able to drink anymore.”*

This deliberate substitution highlights how some participants prioritised alcohol consumption to avoid weight gain associated with food.

### Fear of weight regain leading to choosing alcohol instead

Participants highlighted the psychological struggles associated with the fear of regaining weight after surgery. Jennifer expressed her anxiety about eating more than others in her situation:

*“You start eating, and then I’d be posting saying I’ve just eaten a Tesco meal deal, a sandwich and a bag of crisps. And I’d see other people going, ‘Well, I could barely manage a few mouthfuls after a year.’ And I’m like, Oh my God, what’s wrong with me? I’m back to how I was... my stomach is as big as it was. I’m gonna regain all of my weight, which is a massive fear. So I’d drink instead”*

Denise described how her fear of weight regain led to extreme behaviours, using alcohol to induce sickness:

*“I’d lost the 10 stone; I was looking really good. I didn’t want to put one pound of that 10 stone on, I was terrified of it. Binge drinking and making myself so ill that I couldn’t eat maintained weight loss, and I was addicted to the method. Not realising then what a terrible cycle I was in... It just got to a point where I could eat during the drinking binges, but at the beginning, I just didn’t eat at all. And I were just eternally drunk until I got terribly sick. It’s all about the figures on the scales, and really, it was just sheer dehydration to be honest. But I was absolutely petrified of putting any on.”*

These accounts underscore how the fear of regaining weight can drive unhealthy behaviours, further complicating post-surgery recovery.

### Alcohol as a workaround for eating restrictions

Participants who had a gastric band described difficulties with eating post-surgery and noted that alcohol sometimes helped them manage these challenges. Olivia shared how tightness in the band often made eating problematic, leading her to use alcohol as a workaround:

*“I think of the times where I couldn’t eat because my band was too tight, so I would have a drink instead. It is linked. I drank before, it’s not the band that started me drinking. It’s just that I choose alcohol over food now because it’s easier. You have to try different foods, and you’re on liquids. I quite liked the liquid side because I didn’t have to think about food. I’d go out for a meal, look at the menu, and think, ‘Oh, I’m going to struggle with this. I’ll just have a drink….’”*

Petra similarly used alcohol to help relax, making eating more manageable:

*“The problem with the band is that some days you can’t eat, and some days you can. On the days you can’t, sometimes you just pour, and that’s the issue…With alcohol, I can eat better. If I’m stressed, nothing’s going to go down, but with a glass of wine, I’m more relaxed, and the food goes down.”*

Towards the end of her interview, Petra reflected on how alcohol became a convenient solution for busy days:

*“You haven’t always got time to sit there for half an hour to eat a meal. Sometimes it’s just easier to pinch a chip off someone’s plate and wash it down with something that makes it go easy.* *”*

These accounts illustrate how the challenges of eating with a gastric band may lead individuals to turn to alcohol as a substitute or facilitator. While these may appear to individuals initially adaptive, they potentially contribute to problematic drinking behaviours.

**Theme 3: Social dynamics of alcohol use**

### Confidence, ego and social reinvention

Participants highlighted how weight loss post-surgery boosted their confidence and ego, leading to increased socialising and alcohol consumption. Debbie described the newfound sense of self-confidence and its impact on her lifestyle:

*“I'm now size 8 squeezing myself into the smallest tightest dresses ever because I've never had that experience. Then I'm on a dating scene, internet dating, meeting people for drinks, and suddenly I was matching with people who wouldn’t have looked at me before. It gave me a massive ego boost.”*

Similarly, Claire shared:

*“The ego took over because I felt like I was a million dollars. I was going out a lot, wearing smaller clothes, and acting like I was in my 20 s again. I felt sexually liberated, but my behaviour became reckless drinking heavily, taking class A’s. I completely lost the plot, and addiction took off.”*

### From eating to drinking: socialising after weight loss

Participants discussed how weight loss led to more social opportunities, often centred around drinking rather than eating.

Debbie explained:

*“Meals started being replaced by going for drinks as a way to socialise. Friends would ask, ‘Do you want to get something to eat?’ and I’d say, ‘How about a drink instead?’ It became my go-to, but then it turned into something sinister.”*

Julie echoed this, describing how weight loss increased her social interactions:

*“After surgery, I pretty much accepted every invitation. It meant new dresses and going out more. But we’d be walking through the village, and I’d want to stop in every bar we passed. I still wanted to drink more, and my husband would be dragging me home.”*

### Friendship shifts around drinking

Participants noted shifts in their social circles, often gravitating toward friends who matched their new drinking habits while distancing themselves from those who expressed concern. Debbie shared:

*“A year after surgery, people started noticing my drinking. They’d say, ‘You’re drinking a lot,’ but I’d dismiss it as just having fun. I began pulling away from people who voiced concerns because I didn’t want them to intervene.”*

Julie recalled forming a bond with a fellow bypass patient over shared drinking habits:

*“I made friends with a woman who had also had a bypass. We’d drink wine and get drunk quickly while others in the group seemed fine. Tragically, she later died of cirrhosis, and it made me reflect on our shared choices.”*

### Secrecy and social isolation

Participants admitted to concealing their alcohol use, like the secrecy around food consumption pre-surgery. Amy reflected:

*“(Before surgery) After a night out, I’d come home and think, ‘Now I can have something else to eat where nobody can see me.’ It became the same with alcohol. I felt embarrassed going to the same shop repeatedly, so I started rotating shops to buy alcohol.”*

Debbie admitted to hiding the extent of her drinking:

*“There’s no way I would drink the way I do in secret out in public. It’s different when no one’s watching.”*

Claire described how her growing reliance on alcohol led to self-isolation:

*“I realised things were spiralling out of control, so I stopped going out to drink. But then it became about drinking alone. I wanted alcohol more than I wanted people.”*

Taken together, these accounts highlight how weight loss and altered social dynamics can lead to increased alcohol consumption, altered friendships, secrecy, and shifts in social behaviours, often exacerbating problematic drinking patterns.

**Theme 4: Alcohol as a mechanism for emotional coping and stress relief**

### Using alcohol as a coping mechanism

Participants revealed that they turned to alcohol to manage emotions such as grief or stress, a behaviour that previously involved food. Jennifer explained how alcohol replaced food as a coping tool:

*“I could no longer rely on food as a coping mechanism or as a form of escape. Alcohol very quickly took that place. I couldn’t eat, and I didn’t want to sit with uncomfortable feelings. So, food was taken off the table, pardon the pun, and alcohol replaced it. It’s hard for people to understand how losing weight so quickly messes with your head. People say, ‘You look amazing,’ and you’re thinking, ‘Why don’t I feel amazing?’”*

Petra echoed this, linking her alcohol use to stress:

*“Last year, my dad was in hospital, and it was incredibly stressful. I wasn’t eating, didn’t feel hungry, and didn’t need to eat. So, the first thing I’d do when I came home was pour a glass of wine, before I even took my coat off.”*

Julie reflected on how stress would have been managed differently pre-surgery:

*“Before surgery, I’d have turned to a takeaway when stressed. After surgery, it was a glass of wine after work. Wine became a substitute for the emotional comfort food used to provide.”*

### Adverse childhood experiences

Participants reflected on past traumas, including adverse childhood experiences (ACEs), that may have influenced their struggles with food or alcohol. Five participants shared histories involving alcoholism in the family, adoption, divorcee, or bullying. Claire recounted her early experiences:

*“My childhood was filled with massive trauma and neglect. From a young age, food became my comfort, and I was constantly put on diets. The whole diet culture and my relationship, or lack of it, with my mother compounded the problem. I was adopted, which added to the layers of trauma.”*

Denise revealed a difficult childhood dynamic:

*“My mum would medicate me with lorazepam when I was about 10 or 11 because I was a high-maintenance child. It wasn’t right, but she did it. I’ve struggled with insomnia my whole life as a result.”*

Lucy, whose alcohol concerns surfaced years after surgery, linked her avoidance of alcohol to her upbringing:

*“My dad was an alcoholic throughout my childhood. He quit 15 years ago, but by then, I’d already left home. Now, he’s what they call a dry drunk, still as unpleasant sober as he was drunk because he never sought therapy or help for his addiction.”*

These narratives reveal how unresolved emotional issues, often rooted in childhood, intersect with post-surgery vulnerabilities, leading some to use alcohol as a coping mechanism. This points to a shared underlying cause between disordered eating and alcohol use, with both serving as strategies to manage emotional distress.

**Theme 5: Assessment, follow-up and peer support in alcohol related challenges**

### Identifying pre-surgery alcohol use

Participants reflected on their pre-surgery alcohol habits, with six of the eleven acknowledging regular consumption but not identifying it as problematic at the time. Jennifer expressed concern that her behaviours should have raised red flags during pre-surgery assessments:

*“I remember telling the clinician I could sit home on weekends and drink two bottles of prosecco with a large pizza. Looking back, that should have been a warning sign for her.”*

Denise similarly noted that alcohol use was not adequately explored, despite her admitting to drinking 1-2 bottles of wine multiple times a week including the night before her surgery:

*“The night before my surgery, I had two bottles of wine, which should have been a clue. Six weeks later, I tried half a glass and felt fine, so I thought, ‘I’m good to go.’”*

These accounts suggest that potentially problematic alcohol use may have been under-recognised prior to surgery, reflecting limitations in pre-operative screening processes rather than the absence of risk.

### Pre- and post-surgery guidance

Participants described limited information provided about the risks of alcohol use post-surgery, including addiction transfer. Seven participants stated that clinicians only advised them to abstain from alcohol for 6–12 months, without further explanation. Julie believed the guidance was solely due to alcohol’s calorie content:

*“I thought they were just worried about the calories in alcohol affecting the surgery's success. They never explained it was about how my body would process alcohol differently.”*

Lucy, who abstained from alcohol for years post-surgery, found this advice insufficient:

*“My friends bought me a case of wine... There was no discussion about the risks beyond the first year. Knowledge is power, if I’d understood, I could have made informed decisions.”*

Debbie highlighted the lack of pre-surgery information and warned of the risks posed by altered alcohol metabolism:

*“I wasn’t told that alcohol would stay in my body longer. Now I know I have to be extra cautious about drinking and driving because I might not be fine to drive when I think I am.”*

Participants expressed that proper education about these risks could have prevented issues with alcohol. Denise emphasised the importance of raising awareness:

*“My mission now is to warn others about how alcohol can take over post-surgery. People need to consider this before undergoing the procedure.”*

### Sharing personal experiences

Participants highlighted the importance of sharing personal experiences to educate others considering surgery. Jennifer noted that while online weight-loss communities were supportive, they rarely discussed alcohol issues:

*“I mentioned once on Instagram that alcohol post-surgery needs to be talked about, and people agreed, but it wasn’t something that came up much in the community.”*

Petra expressed guilt over inspiring others to have bariatric surgery without knowing the risks:

*“I feel responsible. People saw my success and decided to get banded, but now I know three of them have developed alcohol issues. It’s heartbreaking.”*

These accounts underscore the need for open conversations about alcohol risks in these patient communities and during clinical consultations.

### Counselling and support post-surgery

Participants discussed the lack of effective post-surgery support for addressing alcohol issues. Maria shared her disappointment with the counselling she received:

*“The surgeon and dietician just said I was consuming too many calories through alcohol and needed help. The counselling felt like just talking, and I wasn’t honest about my drinking because I felt ashamed. It didn’t work for me.”*

Denise, awaiting a liver transplant, described receiving no advice from her clinical team:

*“The clinic never discussed alcohol risks, even during my regular check-ups. It wasn’t until my health deteriorated that I sought help. AA and the 12-step programme have been lifesaving.”*

Many participants emphasised that effective follow-up care should include routine mental health assessments, addiction screenings, and accessible counselling tailored to post-surgery needs. Jennifer’s experience with an alcohol agency highlights gaps in available support:

*“They signed me up for harm reduction classes, but I knew I needed total abstinence. My GP couldn’t help because they weren’t alcohol specialists. The system isn’t set up to address these issues for people like me.”*

These reflections underscore the need for better pre- and post-surgery assessments, education on alcohol risks, and structured follow-up care to support patients in navigating these challenges.

## Discussion

This study contributes new understanding to the growing body of research on alcohol use following bariatric surgery, particularly among women. Participants varied in surgery type, time since surgery, and current alcohol use status; however, the analysis focused on shared psychosocial processes rather than subgroup comparison, which was not feasible given the size and heterogeneity of the sample. Individual circumstances (e.g., procedure type or current alcohol use) did not consistently map onto distinct experiential patterns and were therefore not treated as analytic categories. Although prior studies have identified increased rates of post-operative AUD after procedures such as Roux-en-Y gastric bypass (King et al., 2012; Svensson et al., 2013), our findings offer novel insight into why alcohol becomes a problematic coping strategy by centring lived experience and underexplored psychosocial mechanisms.

Consistent with prior research, participants reported heightened sensitivity to alcohol post-surgery due to altered metabolism. Emerging evidence suggests that bariatric surgery may also alter gut–brain signalling and reward processing, potentially heightening sensitivity to alcohol’s reinforcing effects (Blackburn et al., 2017). These biological changes may amplify psychosocial vulnerabilities, including emotional regulation difficulties, making alcohol a particularly potent coping strategy post-surgery. However, our findings deepen this understanding by showing that physiological changes alone do not account for post-operative alcohol use. Instead, participants described conscious use of alcohol as a tool, to suppress appetite, avoid weight regain, or manage social anxiety and emotional distress. These motivations suggest that alcohol serves instrumental, not incidental functions, challenging the notion that addiction transfer is purely unconscious (Brunault et al., 2015; Yoder et al., 2018).

Several themes identified in this study align with the emerging concept of *Food and Alcohol Disturbance*, which describes the substitution of food restriction with alcohol use following bariatric surgery (Khantzian & Albanese, 2010). Participants’ accounts of appetite suppression, calorie trade-offs, and using alcohol for emotional regulation resonate with this framework. However, rather than applying this concept deductively, our analysis highlights how these behaviours may function as contextually embedded coping strategies shaped by trauma, identity shifts, and post-surgical social change.

This aligns with the self-medication hypothesis (Khantzian & Albanese, 2010), but with a critical difference: participants’ behaviours reflect deliberate strategies of emotional and weight control, underscoring a complex interplay of agency, trauma, and societal pressures post-weight loss. Participants’ accounts suggest an interconnected relationship between mood, food, and alcohol, in which post-surgical emotional shifts reinforced alcohol use over food for coping, weight control, or social confidence. This builds on recent critiques of the “addiction transfer” model as overly deterministic and calls for a more nuanced understanding of addiction as a psychosocial adaptation (Esin et al., 2025; Riedel et al., 2024).

There is emerging evidence that challenges deterministic “addiction transfer” models in favour of a shared, modifiable vulnerability framework. Real-world studies of individuals treated with GLP-1 receptor agonists suggest that modulation of gut–brain reward pathways may dampen multiple appetitive behaviours simultaneously, rather than displacing addictive patterns from food to alcohol (Lähteenvuo et al., 2025; Qeadan et al., 2025). Importantly, alcohol-related risk is not uniform across procedures or individuals, and not all patients experience escalation in use following surgery; emerging evidence of polysubstance risk (e.g., opioids) further supports the need for bio-psycho-social models that move beyond assumptions of inevitable transfer.

The findings also illuminate how post-surgery weight loss alters social dynamics and identity, with increased social confidence often linked to risky environments and behaviours. Participants described a trajectory from initial empowerment and social reintegration to eventual dependence, secrecy, and isolation. This challenges conventional post-surgical narratives that frame psychosocial outcomes as uniformly positive (Qeadan et al., 2025) and instead reveals a dialectical process of visibility, vulnerability, and relational change. Similar trajectories have been noted in studies of gender, stigma, and post-surgical adjustment (Conason et al., 2013).

Importantly, this study confirms that psychological and trauma histories play a central role in shaping post-surgery substance use, especially among women. Several participants linked their alcohol use to childhood adversity, reinforcing calls for trauma-informed approaches in bariatric assessment and follow-up care (Leung et al., 2021; Meule et al., 2020). These findings suggest that standard risk screening, which often focuses on current substance use, may overlook hidden vulnerabilities linked to trauma, disordered eating, and emotional regulation difficulties.

Despite emerging calls for integrated mental health care (de Zwaan et al., 2020), most participants in this study received minimal pre-operative psychological evaluation and described fragmented or absent post-operative support. This echoes systemic critiques that bariatric pathways remain overly focused on weight loss outcomes at the expense of holistic recovery (Nafie et al., 2023; Rubino et al., 2020). Beyond psychosocial harm, alcohol use following bariatric surgery may exacerbate nutritional deficiencies and contribute to more severe outcomes, including alcohol-related morbidity and mortality, particularly when alcohol is used for appetite suppression or weight control in the context of limited post-operative support.

Our findings further highlight that informal peer groups and social media can both reinforce harmful behaviours (e.g., drinking to maintain weight loss) and offer limited but critical sources of validation. Participants expressed guilt and responsibility for unintentionally influencing others to pursue surgery without understanding its full psychosocial risks. This underscores the need for structured peer support that is clinically informed and safely moderated (Voigt et al., 2022).

As a qualitative study, the aim was not demographic representativeness but analytic insight into psychosocial processes that may be relevant beyond this sample and transferable to similar clinical contexts.

### Clinical implications

These insights have actionable implications for practice. Pre-surgical pathways must incorporate psychosocial risk screening tools that assess prior trauma, disordered eating patterns, and coping strategies, not just current substance use. For example, established alcohol screening tools (e.g., the AUDIT) (Babor et al., 2001). could be complemented by brief psychosocial assessment focusing on trauma history, coping strategies, and emotional regulation to better identify individuals at risk of post-surgical alcohol-related harm.

While alcohol and substance use are sometimes addressed within pre-surgical psychological assessments in bariatric care, our findings suggest that current screening practices may not adequately capture psychosocial vulnerabilities such as trauma history, emotional regulation difficulties, or the functional use of alcohol as a coping strategy. Brief complementary tools assessing these domains could enhance existing assessments without requiring wholesale changes to current pathways.

Standardised education about post-operative alcohol sensitivity and long-term risks should be mandatory and delivered in gender- and trauma-sensitive formats. Post-operatively, patients would benefit from structured psychosocial follow-up, including access to therapists trained in addiction and bariatric psychology, ideally for at least two years post-surgery. The integration of peer-led, clinician-supported recovery groups could also bridge gaps in the current care system, especially in under-resourced areas. For example, trauma-informed assessment could be embedded within routine care through brief standardised screening delivered pre-operatively and revisited at key transition points (e.g., 6–12 months post-surgery), alongside flexible, clinician-led check-ins during existing follow-up appointments.

### Contributions and future research

This study advances the literature by illustrating that post-surgical alcohol use is not solely a matter of physiological change or unconscious transfer, but often reflects contextually embedded strategies for control, connection, or avoidance. As this study focused on alcohol use and excluded individuals with pre-existing AUD, it may not capture broader patterns of addiction transfer following bariatric surgery. Future research should explore whether the psychosocial processes identified here also underpin transitions to other substances, including polysubstance use reported post-surgery.

Emerging evidence from pharmacological studies, including work on GLP-1 receptor agonists, suggests that shared reward pathways may be modifiable, with addictive patterns potentially dampened rather than displaced. Future research could build on this by integrating psychosocial and biological perspectives to test shared vulnerability models across different bariatric populations (Lähteenvuo et al., 2025; Qeadan et al., 2025).

Further work should explore these dynamics across more diverse samples, including men and ethnically minoritised groups, and examine how online communities shape both awareness and risk. Quantitative longitudinal studies and technology-enabled monitoring may help map alcohol use trajectories and identify opportunities for earlier, personalised intervention following bariatric surgery.

### Limitations

This study is based on a small, self-selected sample of UK-based women, which may limit the generalisability of findings to broader or more diverse populations, including men or those from different cultural backgrounds. All participants were White British or European women recruited via Facebook groups, which may shape the experiences represented in this study. Our recruitment route may privilege the voices of individuals with digital access, engagement in bariatric-related online communities, and potentially more salient or severe concerns about alcohol use. Findings should therefore be understood in terms of analytic transferability rather than demographic representativeness.

Interviews were conducted via Zoom, which may have limited access to non-verbal cues and posed barriers for individuals with limited digital access or confidence. However, remote interviews also enabled participation across a wide geographical area and allowed participants to engage from familiar, private settings, which may have supported disclosure when discussing sensitive experiences.

The study did not examine whether themes differed by surgery type, time since surgery, or current alcohol use status. In particular, physiological differences associated with Roux-en-Y gastric bypass may interact with psychosocial processes in ways not captured here. Future research with larger samples is needed to explore these potential variations.

Data were generated through semi-structured interviews without triangulation from clinical records or clinician perspectives. Findings therefore reflect self-reported accounts of sensitive experiences and may be subject to recall or social desirability bias. Analytic credibility was supported through reflexive thematic analysis, collaborative interpretative dialogue, and co-design with individuals with lived experience.

Exclusion criteria were based on self-reported absence of a formal AUD diagnosis at the time of surgery. As such, some participants may have had pre-surgical patterns of problematic drinking that were not recognised or diagnosed at the time, which should be considered when interpreting findings.

In addition, the inclusion of participants who underwent procedures not consistently associated with increased alcohol-related risk (e.g., gastric banding) means findings should not be interpreted as procedure-specific but rather highlight psychosocial mechanisms that may operate independently of surgical physiology.

Despite these limitations, the study provides valuable insight into underexplored psychosocial processes shaping alcohol use following bariatric surgery.

## Data Availability

The data that support the findings of this study are available from the corresponding author, (KVW) upon reasonable request.
